# Screening of the Pan-African Natural Product Library Identifies Ixoratannin A-2 and Boldine as Novel HIV-1 Inhibitors

**DOI:** 10.1371/journal.pone.0121099

**Published:** 2015-04-01

**Authors:** Ian Tietjen, Fidele Ntie-Kang, Philip Mwimanzi, Pascal Amoa Onguéné, Margaret A. Scull, Thomas Oyebode Idowu, Abiodun Oguntuga Ogundaini, Luc Mbaze Meva’a, Berhanu M. Abegaz, Charles M. Rice, Kerstin Andrae-Marobela, Mark A. Brockman, Zabrina L. Brumme, David Fedida

**Affiliations:** 1 Department of Anesthesiology, Pharmacology, and Therapeutics, University of British Columbia, Vancouver, BC, Canada; 2 Faculty of Health Sciences, Simon Fraser University, Burnaby, BC, Canada; 3 Department of Chemistry, Chemical and Bioactivity Information Centre, Faculty of Science, University of Buea, Buea, Cameroon; 4 Department of Chemistry, Faculty of Science, University of Douala, Douala, Cameroon; 5 Laboratory of Virology and Infectious Disease, The Rockefeller University, New York, NY, United States of America; 6 Department of Pharmaceutical Chemistry, Faculty of Pharmacy, Obafemi Awolowo University, Ile-Ife, Nigeria; 7 African Academy of Sciences, Nairobi, Kenya; 8 Department of Biological Sciences, University of Botswana, Gaborone, Botswana; 9 Department of Molecular Biology and Biochemistry, Faculty of Science, Simon Fraser University, Burnaby, BC, Canada; 10 British Columbia Centre for Excellence in HIV/AIDS, Vancouver, BC, Canada; Helmholtz Zentrum Muenchen—German Research Center for Environmental Health, GERMANY

## Abstract

The continued burden of HIV in resource-limited regions such as parts of sub-Saharan Africa, combined with adverse effects and potential risks of resistance to existing antiretroviral therapies, emphasize the need to identify new HIV inhibitors. Here we performed a virtual screen of molecules from the pan-African Natural Product Library, the largest collection of medicinal plant-derived pure compounds on the African continent. We identified eight molecules with structural similarity to reported interactors of Vpu, an HIV-1 accessory protein with reported ion channel activity. Using *in vitro* HIV-1 replication assays with a CD4+ T cell line and peripheral blood mononuclear cells, we confirmed antiviral activity and minimal cytotoxicity for two compounds, ixoratannin A-2 and boldine. Notably, ixoratannin A-2 retained inhibitory activity against recombinant HIV-1 strains encoding patient-derived mutations that confer resistance to protease, non-nucleoside reverse transcriptase, or integrase inhibitors. Moreover, ixoratannin A-2 was less effective at inhibiting replication of HIV-1 lacking Vpu, supporting this protein as a possible direct or indirect target. In contrast, boldine was less effective against a protease inhibitor-resistant HIV-1 strain. Both ixoratannin A-2 and boldine also inhibited *in vitro* replication of hepatitis C virus (HCV). However, BIT-225, a previously-reported Vpu inhibitor, demonstrated antiviral activity but also cytotoxicity in HIV-1 and HCV replication assays. Our work identifies pure compounds derived from African plants with potential novel activities against viruses that disproportionately afflict resource-limited regions of the world.

## Introduction

While recent advances in antiretroviral therapies (ARVs) have converted HIV to a chronic, manageable condition in many high-income settings, barriers remain for their successful use in low and middle-income countries with high disease burden; for example in parts of sub-Saharan Africa. Here, despite recent significant progress to improve ARV access globally [1], the challenges are complex and can include the fragility and uncertainty of ARV supply systems, limited health care infrastructure, and the ability to retain patients in care [2, 3]. Moreover, the side-effects of ARVs can result in poor adherence and increased risk of viral resistance [3, 4]. Drug resistance has been documented to all licensed ARVs [5], and transmission of resistant HIV remains a major concern in many sub-Saharan African nations [6]. Thus new HIV therapies that target additional viral proteins and are derived from local sources may be particularly advantageous in these regions.

Natural products are a promising but undervalued resource for identifying new antivirals [2]. Compounds derived from these sources can encompass structural diversity that falls outside the scope of chemical spaces found in many synthetic chemical screening libraries [1, 7]; as such, they have the potential to act via mechanisms distinct from those of conventional therapies. With this advantage in mind, the pan-African Natural Product Library (p-ANAPL) was formed to provide a centralized resource of pure compounds obtained from local plants with medicinal properties supported by indigenous knowledge [8]. To date, the p-ANAPL contains over 500 pure compounds and represents the largest physical collection of natural products from medicinal plants in Africa [9]. The p-ANAPL thus represents an opportunity to screen for new inhibitors of pathogens that disproportionately affect countries on the African continent.

**Fig 1:**
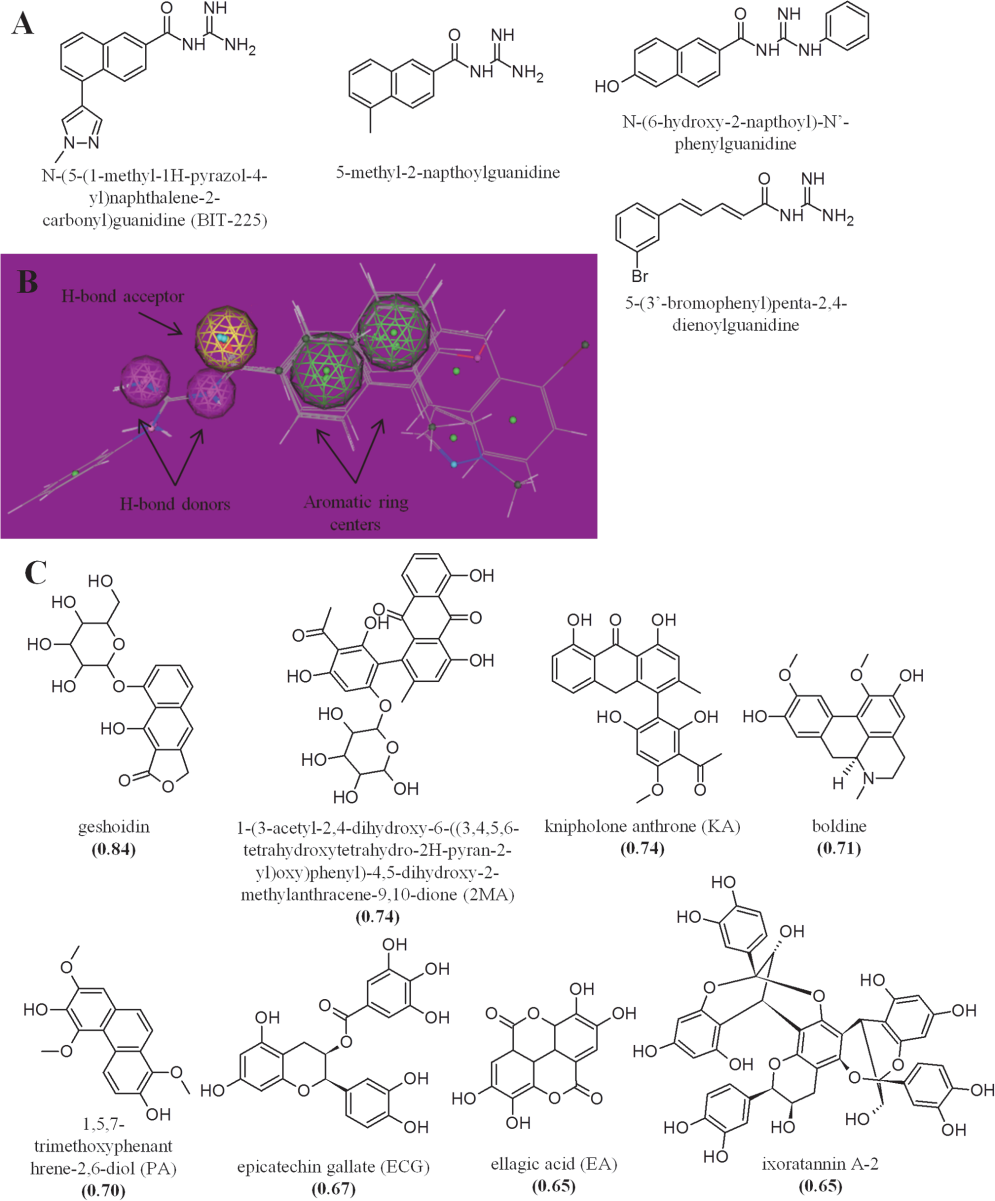
Putative HIV-1 Vpu inhibitors identified from virtual screening of the p-ANAPL. **A,** Structures of four molecules predicted to interact with the Vpu ion channel [20]. **B,** Alignment of four molecules. Chemical substituents that define a shared pharmacophore are highlighted. **C,** Eight p-ANAPL molecules containing aspects of the shared pharmacophore. For each compound, root mean square deviation (RMSD) values are shown in parentheses.

Currently, no licensed ARVs target the accessory proteins of HIV-1. Vpu is an 81–82 amino acid transmembrane protein that is found in HIV-1 and a subset of SIVs and enhances viral replication through multiple functions [10, 11]. Specifically, Vpu augments virion release by downregulating CD4 and the host restriction factor BST2/CD317/tetherin, which otherwise captures mature virions at the cell surface. While HIV-1 with defective Vpu can replicate *in vitro* in some cell lines, these viruses generally display reduced spread, defects in viral budding, and accumulation at the surface of infected cells. Consistent with these phenotypes, a Vpu-deficient chimeric SIV/HIV strain replicated poorly and did not cause depletion of CD4+ lymphocytes in macaques [12]. Thus, effective replication of HIV *in vivo* requires a functional Vpu protein, which makes it a promising drug target. Vpu is also reported by some to have ion channel activity [13–15], although this is controversial [16] and the role of this activity in HIV-1 replication remains uncertain [11]. Nevertheless, a few compounds with an acylguanidine moiety including hexamethylene amiloride and BIT-225 (Fig. 1A) are reported to inhibit both Vpu ion channel activity in lipid bilayers and viral replication *in vitro* [11, 17]. However, the cellular toxicity often associated with this class of compounds is an obstacle toward their further development as antivirals [18, 19].

Here we performed a virtual screen of the p-ANAPL to identify molecules with structural similarity to BIT-225 and other reported Vpu ion channel interactors, with the goal of identifying novel inhibitors of HIV-1. Of eight compounds identified in this virtual screen, both ixoratannin A-2 and boldine displayed antiviral activity in cell-based HIV-1 and hepatitis C virus (HCV) replication assays. We further investigated the mechanisms of action of these antiviral compounds using recombinant HIV-1 strains that either lacked Vpu or encoded patient-derived sequences resistant to established ARVs.

## Materials and Methods

### Chemicals, cells, and reagents

Boldine, ECG, EA, and rimantadine hydrochloride were purchased from Sigma. Geshoidin, 2MA, KA, PA, and ixoratannin A-2 were obtained as previously described [21–25]. CEM-GXR and Huh-7.5 cells were cultured and infected as described previously [26, 27].

BIT-225 was synthesized as previously reported [28]. ^1^H and ^13^C NMR spectra were recorded on a Bruker Ascend spectrometer at 400 and 100 MHz, respectively. Proton NMR data were reported as multiplicities: s for singlet, d for doublet, dd for double of doublets, t for triplet, q for quartet, br s for broad singlet, and m for multiplet. Chemical shifts were reported in parts per million (ppm) and coupling constants in hertz (Hz). For ^1^H NMR spectra, CDCl_3_, or DMSO-d_6_ was used as solvent and served as the internal standard at δ 7.26, or 2.54, respectively. For ^13^C NMR spectra, multiplicities were established by DEPT experiments and CDCl_3_ or DMSO-d_6_ was used as solvent, which served as the internal standard at δ 77.16 (CDCl_3_), or 39.52 (DMSO-d_6_). The purity of each analogue was confirmed by HPLC analysis and using a Finnigan Surveyor HPLC system (Thermo Scientific) consisting of a solvent degasser, a quaternary pump and a Surveyor Auto-sampler Plus module. Chromatographic separation was achieved using gradient elution system consisting of 10–60% acetonitrile in distilled water containing either 5mm ammonium acetate or 0.1% acetic acid. Solvent was pumped through a C18 analytical column (HALO C18 2.7 μM, 4.6 X 50 mm) and delivered at 1.2–2.0 ml/min over 5 min. The column was maintained at room temperature using a column oven. All compounds were dissolved in methanol and detected using a variable wavelength UV detector at 254 nm. The purity was calculated from an integral of peaks and described in terms of percentage (%). Low-resolution mass spectra were obtained using Waters ZQ instrument equipped with ESCi ion source with in-like Waters 2695 HPLC system controlled by MassLynx 4.1 software. ^1^H NMR (400 MHz, CDCl_3_): δ 1.91 (s, 2H), 3.90 (s, 3H), 7.76 (dd, J = 1.6 Hz, J = 8.5 Hz, 1H), 7.83 (d, J = 8.5 Hz, 1H), 7.96 (d, J = 8.5 Hz, 1H), 8.0 (s, 1H), 8.1 (s, 1H), 8.15 (dd, J = 1.6 Hz, J = 8.5 Hz, 1H), 8.2 (s, 1H), 8.59 (s, 1H). ^13^C NMR (100 MHz, DMSO-d_6_): 21.5, 122.2, 122.4, 124.9, 126.6, 127.1, 128.8, 128.9, 130.0, 131.2, 131.7, 135.2, 136.1, 136.8, 163.2, 172.5, 175.9. MS (ESI) m/z: [M + H]^+^ Calculated for C_16_H_15_N_5_O: 294.13; Found: 294.4. Purity: >99%.

The following reagents were obtained through the NIH AIDS Reagent Program, Division of AIDS, NIAID, NIH: pNL4-3 from Dr. Malcom Martin (Catalog #114) and p210-13 (HIV-1_Δvpu_) from Dr. Ronald Desrosiers: (Catalog #2484) [29].

### Virtual screening

The chemical structures of four known Vpu blockers were retrieved from the literature [20]. The 3D conformations were generated using the (default) MMFF94x forcefield [30], using the MOE software tool [31], following the protocol previously implemented by Daveu et al. [32]. The force field parameters were kept at their default values of the Strain Limit of 4 kcal/mol and the Conformations limit of 250 conformations/molecule. The other settings were kept at their default values, with the exception of the Split Output option being turned off, and the Input Filters being turned off. A pharmacophore query is created using the Pharmacophore Query Editor, implemented in MOE. A query consists of a set of constraints on the location and type of pharmacophoric features, which can be used to search a database of molecular conformations. In this work, the query was created and saved, in order to be used later in a pharmacophore search. While a query may be created without a reference molecule (*e*.*g*., the most active molecule in our database), in this study, the common pharmacophore features of all four compounds was used in the virtual screening of the p-ANAPL library (exported in .mdb format in MOE) [9]. The Pharmacophore Search was carried out on the p-ANAPL library [9], using the pharmacophore module of the MOE package. Compounds with four out of the five common query features were selected as 'hits' in this search. The enrichment was measured against a database that has already been enriched by a factor of 10 using MACCS fingerprints. The Pharmacophore Consensus provided suggested features given an aligned set of molecules. In this study, features that may contribute to the pharmacophore alignment of the four Vpu blockers were located. How well the pharmacophore features of the hits fitted into the common pharamcophore of Vpu actives was expressed as a root mean square deviation (RMSD), with the compounds with lowest RMSD selected as hits.

### Generation of viruses

HIV-1_Δvpu_ was constructed by digesting plasmid p210-13 3’ Δ-vpu [29] with *Eco*RI and *Nhe*I enzymes. The resulting DNA fragment was then ligated into wild-type pNL4-3 plasmid digested with the same enzymes. HIV-1_Δvpu_ and HIV-1_NL4-3_ viral stocks were prepared by transfection of HEK-293T cells, as previously described [33]. Patient plasma-HIV-1 RNA sequences were analyzed for resistance mutations using the HIV Drug Resistance Database Genotype Resistance Interpretation Algorithm (hivdb.stanford.edu) [5]. Recombinant HIV-1_NL4-3_ expressing patient-derived HIV-1 sequences harboring resistance mutations to protease and reverse transcriptase inhibitors (HIV-1_PR+RT_), reverse transcriptase inhibitors (HIV-1_RT_), and integrase inhibitors (HIV-1_INT_) were generated by homologous recombination following co-transfection as reported [34].

The intergenotypic chimeric HCV used in this study—Bi-Gluc-J4(1b)/JFH1(2a) containing cell culture adaptive mutations in NS2 (T2996C) and NS3 (A4827T)—was previously described [35] and subsequently modified to express Gaussia luciferase in the first cistron. HCV stocks used for infection were generated by electroporation of in vitro transcribed RNA into Huh-7.5.1 cells and pooling of supernatants harvested between 60–96 hr post-electroporation. HCV infectivity was determined by limiting dilution assay as previously described [27].

### HIV-1 replication assays in CEM-GXR cells

CEM-GXR cells are an immortalized CD4+ T-lymphocyte line engineered that naturally express the HIV-1 coreceptor CXCR4 and have been further engineered to express the HIV-1 co-receptor CCR5 and a Tat-driven LTR-GFP expression cassette. As such, intracellular GFP expression indicates HIV-1 infection [26]. Cultures of 10^5^ CEM-GXR cells were seeded into 96-well plates with RPMI-1640 media (Sigma) supplemented with penicillin/streptomycin plus 20% fetal calf serum (R20+), infected with HIV-1 at a multiplicity of infection of 0.03 (to achieve 3% infected cells on Day 2), and incubated at 37°C and 5% CO_2_. 24 hours later, cells were pelleted at 750 g and resuspended in R20+ containing compound or 0.1% DMSO. Stock concentrations of all compounds were prepared in DMSO such that the final concentrations of compounds in culture medium did not exceed 0.1% DMSO. Cells were then incubated at 37°C and 5% CO_2_ for an additional 72 h. 5,000 cells from each culture were measured for cell toxicity and GFP expression by flow cytometry. Flow cytometry data were analyzed using FlowJo v. 8.8.7 software (FlowJo LLC, Ashland, OR). Cell toxicity in HIV-1 infected cell cultures with compound was calculated as the relative percentage of CEM-GXR cells that displayed characteristic forward- and side-scatter parameters compared to control infected cultures plus 0.1% DMSO. Background GFP expression in uninfected cultures was set at 0.05% GFP-positive cells. For each experiment, all data points were obtained in triplicate. All data are reported as the mean ± s.e.m. from at least 3 independent experiments performed with different virus stocks and/or performed on different days. However, all experiments that compare HIV-1_NL4-3_ and mutant HIV-1 were performed in parallel. EC_50_ and CC_50_ values for each compound were calculated from at least four concentrations. Statistical analyses were performed using Student’s unpaired t test, with a p value < 0.05 plus a Bonferroni correction to adjust for multiple comparisons considered significant.

### HIV-1 replication assays in peripheral blood mononuclear cells (PBMCs)

PBMCs from HIV-1 negative subjects, purchased commercially (StemCell Technologies, Vancouver, Canada), were maintained in RPMI-1640 media (Sigma) supplemented with penicillin/streptomycin plus 10% fetal calf serum (R10+). 72 h prior to viral infection, PBMCs were stimulated by addition of 5 μg/mL phytohaemaglutanin (PHA) (Day 0) and maintained in R10+ medium supplemented with 100 units/mL human IL-2. At Day 3 following cellular activation, 3x10^5^ cells were infected with HIV-1_NL4-3_ at a multiplicity of infection of 0.003 (0.3% infected cells) and incubated at 37°C for 6h, followed by wash and re-suspension in fresh R10+ medium supplemented with 100 units/mL human IL-2 and with or without compounds at defined concentrations. Culture supernatants were collected and replaced with fresh medium supplemented with human IL-2 every 3 days (*i*.*e*., Days 6, 9, and 12) with or without fresh compounds. Viral replication at day 12 was monitored by measuring cell culture supernatant levels of p24^Gag^ by ELISA (XpressBio, Frederick, MD) following manufacturer’s instructions.

Cytotoxicity was determined by ViaCount Assay (Millipore). Briefly, Day 3 PHA-activated PBMCs (3*10^5^) were cultured in R10+ supplemented with 100 units/mL human IL-2 in the presence of 0.1% DMSO or compounds at defined concentrations. At Day 5, 15 μL of each PBMC culture was mixed with Guava ViaCount Reagent at a 1:10 dilution. Data were collected using a Guava EasyCyte HT Flow Cytometer (Millipore).

### HCV cell culture, viral replication assays

HCV cell culture was performed as described previously [27]. Briefly, 4*10^4^ Huh-7.5 cells were seeded into 24-well plates and incubated at 37°C and 5% CO_2_ overnight. Cells were then infected with HCV (multiplicity of infection = 0.3) for 6 hours, followed by removal of HCV and incubation with fresh medium containing compounds at stated concentrations. Cultures were then incubated for 72 hours, with no more than 0.1% DMSO present in culture medium. Cells were then trypsinized, resuspended in culture medium, and stained with the 9E10 alexa-fluor-conjugated antibody to NS5A [27]. Cells were fixed and analyzed for NS5A fluorescence by flow cytometry, and data were analyzed using FlowJo software. Cell toxicity in HCV infected cell cultures with compound was calculated as the relative percentage of Huh-7.5 cells that displayed characteristic forward- and side-scatter parameters compared to control infected cultures plus 0.1% DMSO. For each culture, NS5A fluorescence was determined as the percent gated cells relative to percent gated cells in infected cultures plus 0.1% DMSO. All experiments were performed in at least duplicate in at least two independent experiments.

## Results

### Virtual screening for potential Vpu inhibitors

Previously reported ligand docking simulations predict that BIT-225 and three additional acylguanidine-containing molecules interact with the pore of a pentameric bundle formed by Vpu transmembrane helices [20] (Fig. 1A). Superposition of these four structures indicates a common pharmacophore consisting of one or more aromatic rings separated by a short spacer from an H-bond acceptor and one or more H-bond donors (Fig. 1B). On the basis of this pharmacophore, we used a probability densities method [9] to virtually screen the entire p-ANAPL. We then selected molecular configurations that emphasize low strain energy, similar shape to the four reference molecules, and similar overlap of aromatic, donor, and acceptor atoms. From this screen, 8 molecules exhibited features similar to the original pharmacophore, defined here as an RMSD > 0.6 (Fig. 1C).

### Identification of HIV-1 inhibitors

**Fig 2:**
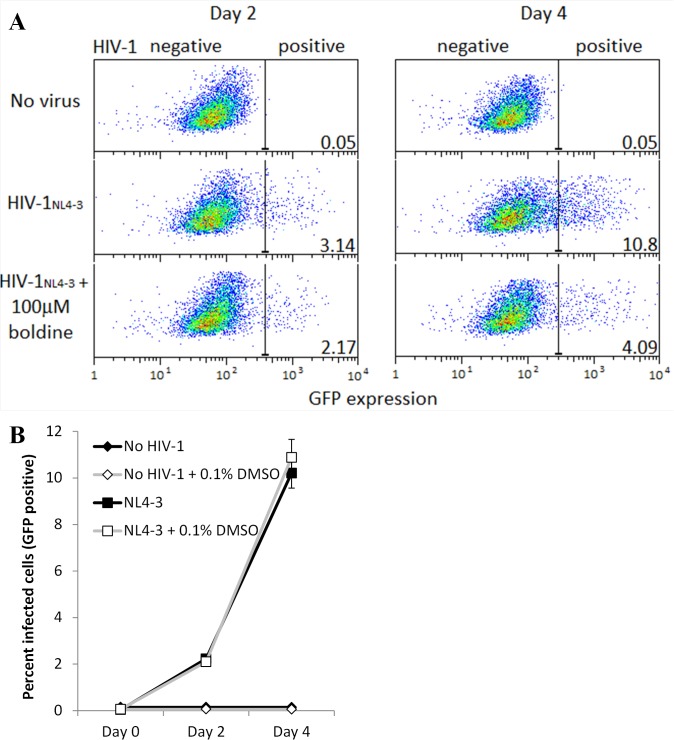
HIV-1 replication in CEM-GXR cells. **A,** Flow cytometry data showing GFP expression from uninfected (top), HIV-1_NL4-3_-infected cells (center), and HIV-1_NL4-3_-infected cells plus 100 μM boldine (bottom) at Days 2 and 4. Values at the bottom-right of each panel indicate the percent GFP-positive (*i*.*e*., HIV-infected) cells. Background GFP expression is set to 0.05% in uninfected cell cultures. **B,** Percent infected cells, as measured by GFP expression, in cultures of CEM-GXR cells +/- HIV-1 and +/- 0.1% DMSO vehicle control. Data in **B** and **A** are from independent experiments.

To assess the effects of these eight compounds on cell viability and HIV-1 replication, we infected a GFP-reporter CEM CD4+ T cell line (CEM-GXR) [26] with the NL4-3 strain of HIV-1 (HIV-1_NL4-3_). Briefly, cells were infected with HIV-1_NL4-3_ at a multiplicity of infection of 0.03 (Day 0), followed by removal of HIV-1 and addition of compound (or 0.1% DMSO vehicle control) 24 hours later (Day 1) and incubation for an additional 72 hours to allow HIV-1 spread (Days 2–4). On Days 2 and/or 4, the percent HIV-1 infected (*i*.*e*., GFP-expressing) cells was measured by flow cytometry. Cell viability was assessed by measuring the percentage of cells displaying normal forward- and side-scatter parameters in drug-treated, HIV-1 infected cultures compared to 0.1% DMSO-treated, HIV-1 infected control cultures. As shown in Fig. 2, the percent infected (GFP-positive) cells in HIV-1_NL4-3_ infected cultures tripled between Day 2 to Day 4 (*e*.*g*., 3.14 to 10.80% in Fig. 2A). HIV-1_NL4-3_ replication was unaffected by the presence of 0.1% DMSO vehicle control (Fig. 2B).

**Fig 3:**
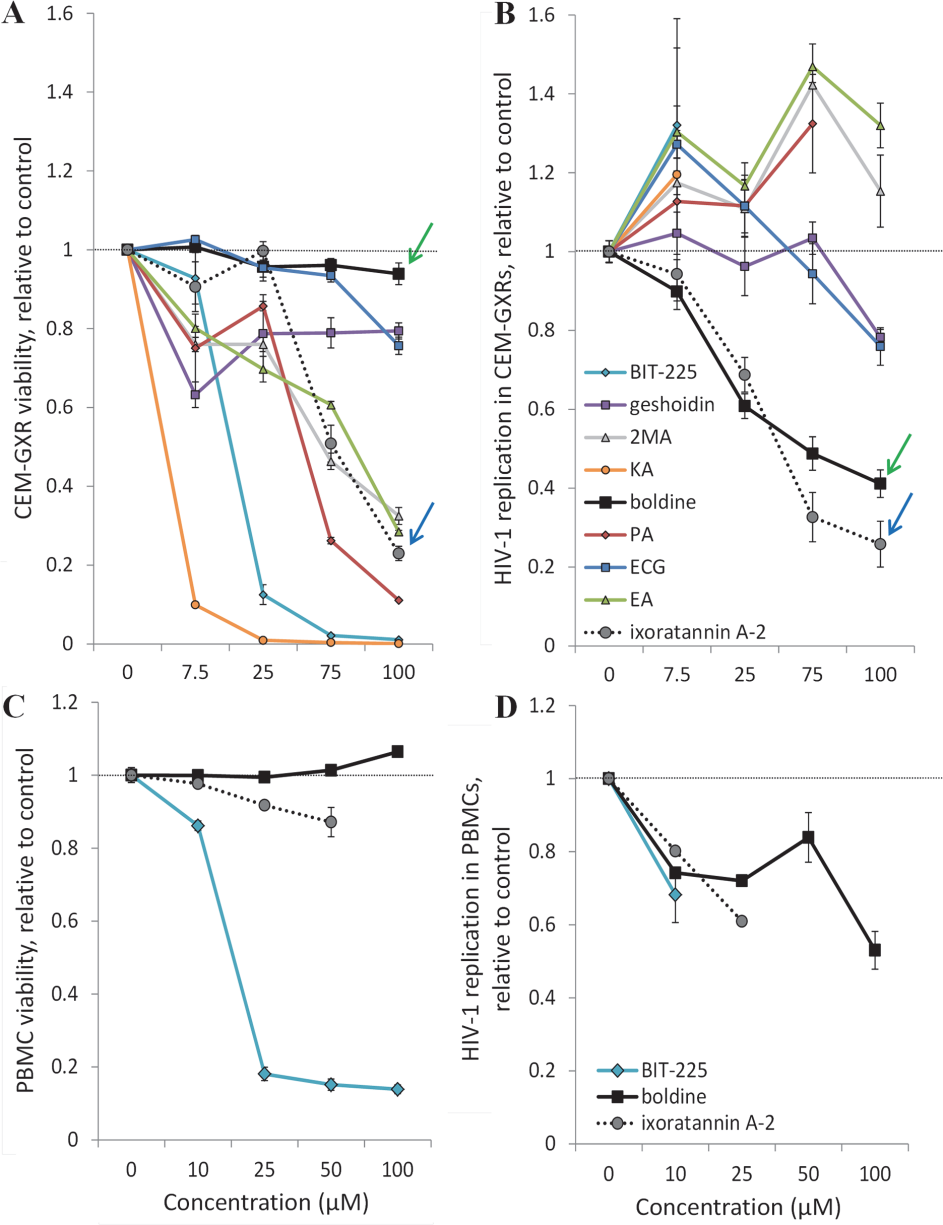
Effects of p-ANAPL compounds on cell viability and *in vitro* HIV-1 replication. **A,** CEM-GXR cell viability at Day 4 in the presence of p-ANAPL compounds at defined concentrations. Data are normalized to the percent of viable cells in an HIV-1_NL4-3_-infected culture plus 0.1% DMSO. **B,** HIV-1 replication in CEM-GXR cells at Day 4 in the presence of compounds. Data are normalized to percent HIV-1_NL4-3_-infected cells plus 0.1% DMSO. Dose-response plots of ixoratannin A-2 and boldine are highlighted with blue and green arrows, respectively. **C**, PBMC viability at Day 5 in the presence of p-ANAPL compounds. Data are normalized to the percent of viable cells in an uninfected culture plus 0.1% DMSO. **D**, HIV-1 replication in PBMCs at Day 12 in the presence of p-ANAPL compounds, as measured by p24^Gag^ levels in cell culture supernatants. Data are normalized to percent HIV-1_NL4-3_-infected cells plus 0.1% DMSO.

Consistent with the previously described cytotoxic effects of Vpu inhibitor BIT-225 in T cell cultures [19, 36], treatment of infected CEM-GXR cells with this compound resulted in substantial cellular toxicity at all tested concentrations (CC_50_ = 10.7 μM; Table 1; Fig. 3A), which obscured the detection of any antiviral activity in this assay. Two naturally-derived compounds, 1,5,7-trimethoxyphenanthrene-2,6-diol (PA) and knipholone anthrone (KA), also displayed cytotoxicity at low concentrations (CC_50_ < 30 μM) and so measures of their antiviral activity were not reliably obtained. However, of the remaining six compounds identified from the virtual screen, two exhibited reproducible inhibition of HIV-1 replication, defined here at an EC_50_ < ~50 μM (Fig. 3B), at concentrations that were not obviously cytotoxic. These compounds were ixoratannin A-2 (CC_50_ = 57.5 μM; EC_50_ = 34.4 μM; selectivity index [SI] = 1.7) and boldine (CC_50_ = 207.7 μM; EC_50_ = 50.2 μM; SI = 4.1; Table 1).

**Table 1:** Cell toxicity and inhibition of HIV-1_NL4-3_ in CEM-GXR cells by p-ANAPL compounds.

Compound	Cell toxicity (CC50, μM)	HIV-1_NL4-3_ inhibition (EC50, μM)
BIT-225	10.7	n/d
geshoidin	>10	>100
2MA	50.9	>100
KA	0.9	n/d
boldine	>100	50.2
PA	26.8	n/d
ECG	>100	>100
EA	52.3	>100
ixoratannin A-2	57.5	34.4

n/d, not determined.

The effects of BIT-225, boldine, and ixoratannin A-2 were also assessed for cell viability and HIV-1 replication in peripheral blood mononuclear cells (PMBCs). Similar to CEM-GXR cells, we also observed substantial cytotoxicity in PBMCs in the presence of BIT-225 (CC_50_ = 12.8 μM; Fig. 3C); in contrast, viability remained above 85% relative to PBMCs in 0.1% DMSO for all assessed concentrations of boldine or ixoratannin A-2. However, a clear reduction in HIV-1 replication in PBMCs, as measured by viral p24^Gag^ protein levels in PBMC culture supernatants, was observed with 10 μM BIT-225 (*i*.*e*., 31.8% reduced replication; Fig. 3D). For boldine, HIV-1 replication was substantially reduced only at high concentrations (47.0% reduced replication at 100 μM), suggesting a roughly 2-fold reduction in activity in PBMCs relative to CEM-GXR cells (Table 1). In contrast, HIV-1 replication in PBMCs in the presence of ixoratannin A-2 roughly corresponded to its activity in CEM-GXR cells (39.0% reduced replication at 25 μM). Unfortunately, due to the limits of available ixoratannin A-2, we were unable to assess its activity in PBMCs concentrations higher than 25 μM.

### Anti-HIV mechanisms of action of ixoratannin A-2 and boldine

To investigate the mechanisms by which ixoratannin A-2 and boldine may block HIV-1 replication, we used a previously-reported HIV-1_NL4-3_ strain lacking the Vpu coding region (HIV-1_Δvpu_) [29]. We also constructed recombinant HIV-1_NL4-3_ strains harboring protease, reverse transcriptase, and/or integrase sequences of HIV-1 strains from patients who had developed resistance to the protease inhibitor Indinavir (IND), the non-nucleoside reverse transcriptase inhibitor Efavirenz (EFV), and/or the integrase inhibitor Raltegravir (RAL) (denoted HIV-1_PR+RT_, HIV-1_RT_, and HIV-1_INT_, respectively). Re-sequencing of the recombinant viruses confirmed the presence of multiple mutations known to confer resistance to IND, EFV, and/or RAL (Table 2) [5].

**Table 2:** Properties of HIV-1 strains lacking Vpu or encoding mutations that underlie resistance to ARVs.

HIV-1 strain	Protein	Primary mutation(s)	Resistance to ARVs tested
Δvpu	vpu	vpu open reading frame deleted	n/a
PR+RT	protease	M46L, I54V, I84V, L90M	IND + EFV
	reverse transcriptase	K101E, Y181C, G190A	
RT	reverse transcriptase	K103N, V108I, M230L	EFV
INT	integrase	N155H	RAL

**Fig 4:**
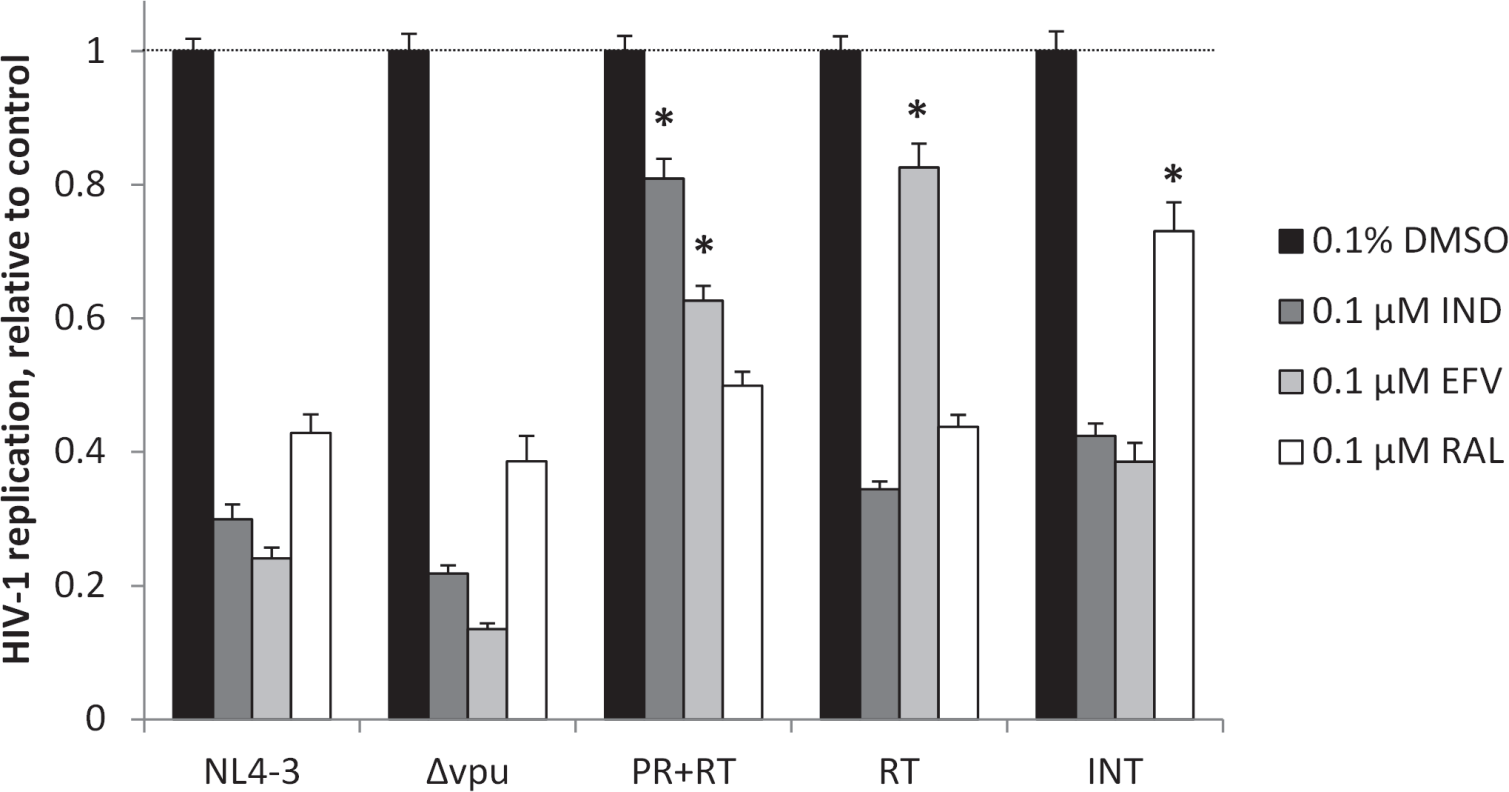
Replication of HIV-1_NL4-3_, HIV-1_Δvpu_, and drug-resistant HIV-1 strains in CEM-GXR cells. Data shown are at Day 4 in the presence of 0.1 μM of established ARVs, relative to the replication rate of each HIV-1 strain in the presence of 0.1% DMSO vehicle control. *, p < 0.004 vs. HIV-1_NL4-3_ replication.

As expected, replication of both HIV-1_NL4-3_ and HIV-1_Δvpu_ in CEM-GXR cells were inhibited in the presence of 0.1 μM EFV, IND, or RAL (Fig. 4). In contrast, HIV-1_PR+RT_, HIV-1_RT_, and HIV-1_INT_ were significantly less sensitive to these drugs in a pattern consistent with specific resistance mutations. For example, HIV-1_PR+RT_ replication was 2.6-fold higher than HIV-1_NL4-3_ in the presence 0.1 μM EFV and 2.7-fold higher in the presence of 0.1 μM IND. Similarly, HIV-1_RT_ replication in CEM-GXR cells was 3.4-fold greater than HIV-1_NL4-3_ replication in the presence of 0.1 μM EFV. Finally, HIV-1_INT_ replication was 1.7-fold higher than HIV-1_NL4-3_ in the presence of 0.1 μM RAL, confirming that these mutations underlie resistance to specific ARVs. The residual replication of HIV-1_NL4-3_ and HIV-1_Δvpu_ in the presence of licensed ARVs to which they are susceptible is likely attributable to our high infection of cell cultures for 24 hours prior to addition of compounds.

**Fig 5:**
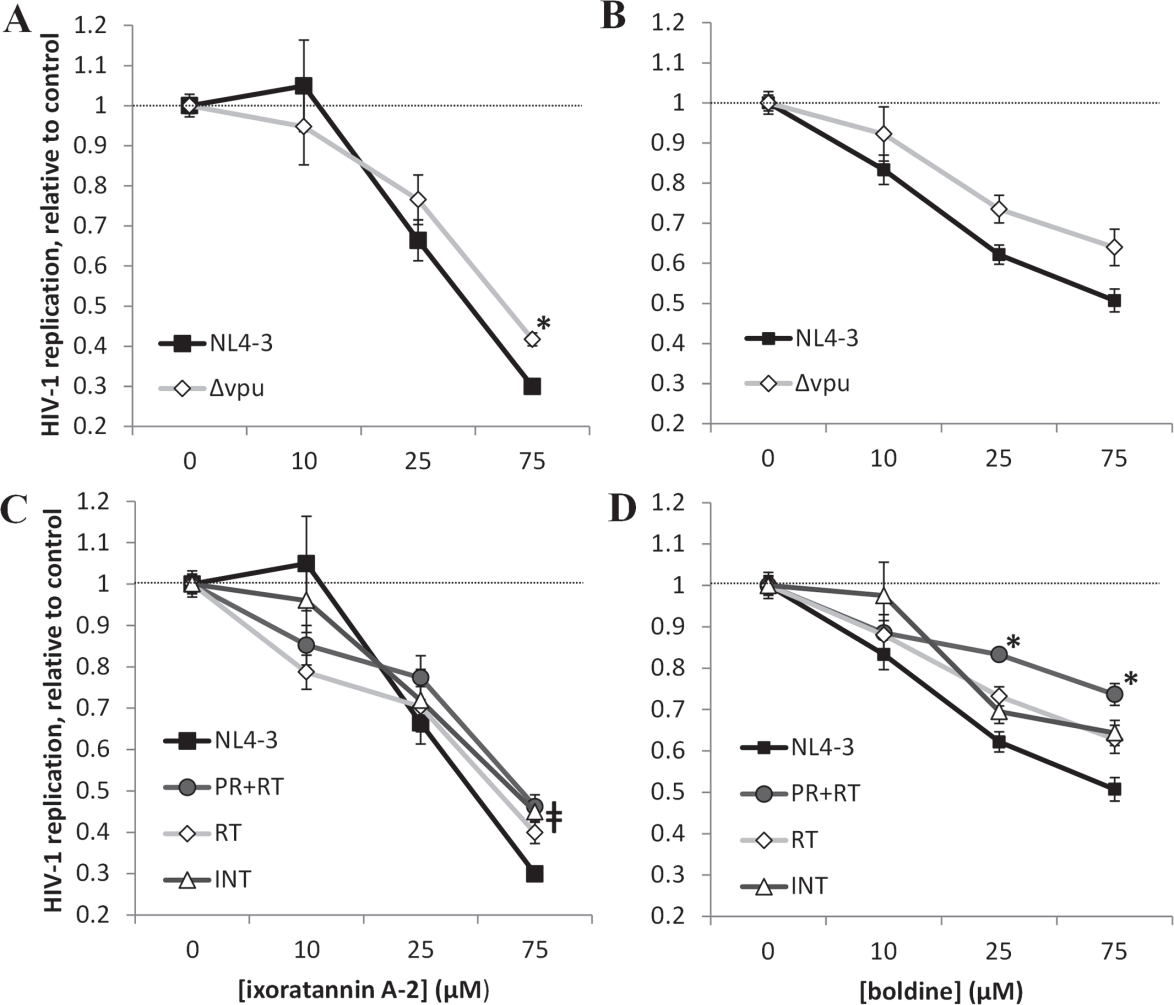
Effects of ixoratannin A-2 and boldine on replication of HIV-1_Δvpu_ and ARV-resistant HIV-1 strains in CEM-GXR cells. In all experiments, data are normalized to the replication rate of each HIV-1 strain plus 0.1% DMSO at Day 4. **A-B,** effects of ixoratannin A-2 (**A**) and boldine (**B**) on HIV-1_NL4-3_ and HIV-1_Δvpu_ replication. **C-D,** effects of ixoratannin A-2 (**C**) and boldine (**D**) on replication of ARV-resistant HIV-1 strains.*, p < 0.008 vs. HIV-1_NL4-3_ at the same concentration of ixoratannin A-2 or boldine. ǂ, p < 0.008 for strain HIV-1_PR+RT_ and HIV-1_INT_ vs. HIV-1_NL4-3_ at same concentration of ixoratannin A-2.

When compared to HIV-1_NL4-3_, 75 μM of ixoratannin A-2 was 1.4-fold less effective at inhibiting HIV-1_Δvpu_ in CEM-GXR cells, a modest difference that was nevertheless statistically significant (Fig. 5A). This difference translated into a 1.5-fold increase in the EC_50_ (EC_50_ = 52.0 μM against HIV-1_Δvpu_ vs. 34.4 μM against HIV-1_NL4-3_; Table 3). This supports Vpu as a direct or indirect target, among possibly others, of ixoratannin A-2. Furthermore, although not reaching statistical significance, treatment with 75 μM of boldine was 1.3-fold less effective at inhibiting HIV-1_Δvpu_ (Fig. 5B), resulting in an EC_50_ > 100 μM compared to 50.2 μM for HIV-1_NL4-3_ (Table 3). This suggests that boldine may primarily confer anti-HIV-1 activity by acting on additional targets other than Vpu.

**Table 3:** Calculated EC_50_s of ixoratannin A-2 and boldine on HIV-1_Δvpu_ and ARV-resistant HIV-1 strains.

Compound	EC50 (μM)				
	HIV-1_NL4-3_	HIV-1_Δvpu_	HIV-1_PR+RT_	HIV-1_RT_	HIV-1_INT_
ixoratannin A-2	34.4	52.0	36.4	33.7	38.4
boldine	50.2	>100	>100	>100	>100

While 75 μM of ixoratannin A-2 was somewhat less effective in blocking replication of HIV-1_PR+RT_ and HIV-1_INT_ in CEM-GXR cells (both exhibited 1.5-fold higher replication than HIV-1_NL4-3_ at this concentration; Fig. 5C), the EC_50_s of ixoratannin A-2 against these resistant HIV-1 strains approximated that of HIV-1_NL4-3_ (Table 3). This suggests that ixoratannin A-2 exhibits comparable activity against wild-type HIV-1_NL4-3_ as well as HIV-1 strains resistant to currently-licensed protease, reverse transcriptase, and integrase inhibitors, except perhaps at very high concentrations. In contrast, treatment with boldine resulted in < 50% inhibition of all ARV-resistant HIV-1 strains at concentrations up to 75 or 100 μM (Fig. 5D), corresponding to EC_50_s > 100 μM for these ARV-resistant strains compared to 50.2 μM for HIV-1_NL4-3_ **(** Table 3). In particular, boldine was less effective at blocking replication of HIV-1_PR+RT_, with 1.4 and 1.5-fold greater replication vs. HIV-1_NL4-3_ at 25 and 75 μM, suggesting that it may act as an HIV-1 protease inhibitor with some cross-resistance to reverse transcriptase and integrase inhibitors.

### Anti-hepatitis C virus activity of ixoratannin A-2 and boldine

As ixoratannin A-2 and boldine potentially acted on multiple HIV-1 targets and exhibited no obvious cellular toxicity, we asked whether these compounds blocked replication of other viruses. We therefore assessed the effects of ixoratannin A-2, boldine, and BIT-225 on replication of HCV, which also encodes a viral ion channel and protease [37]. Using an established cell culture system [27], Huh-7.5 cells were infected with an intergenotypic chimera between the J4 (genotype 1b) and JFH1 (genotype 2a) strain of HCV for 6 hours, followed by incubation with fresh medium containing compounds at defined concentrations. Infected cells were then incubated for an additional 72 hours. NS5A expression, a marker of HCV infection, was then detected by flow cytometry.

**Fig 6:**
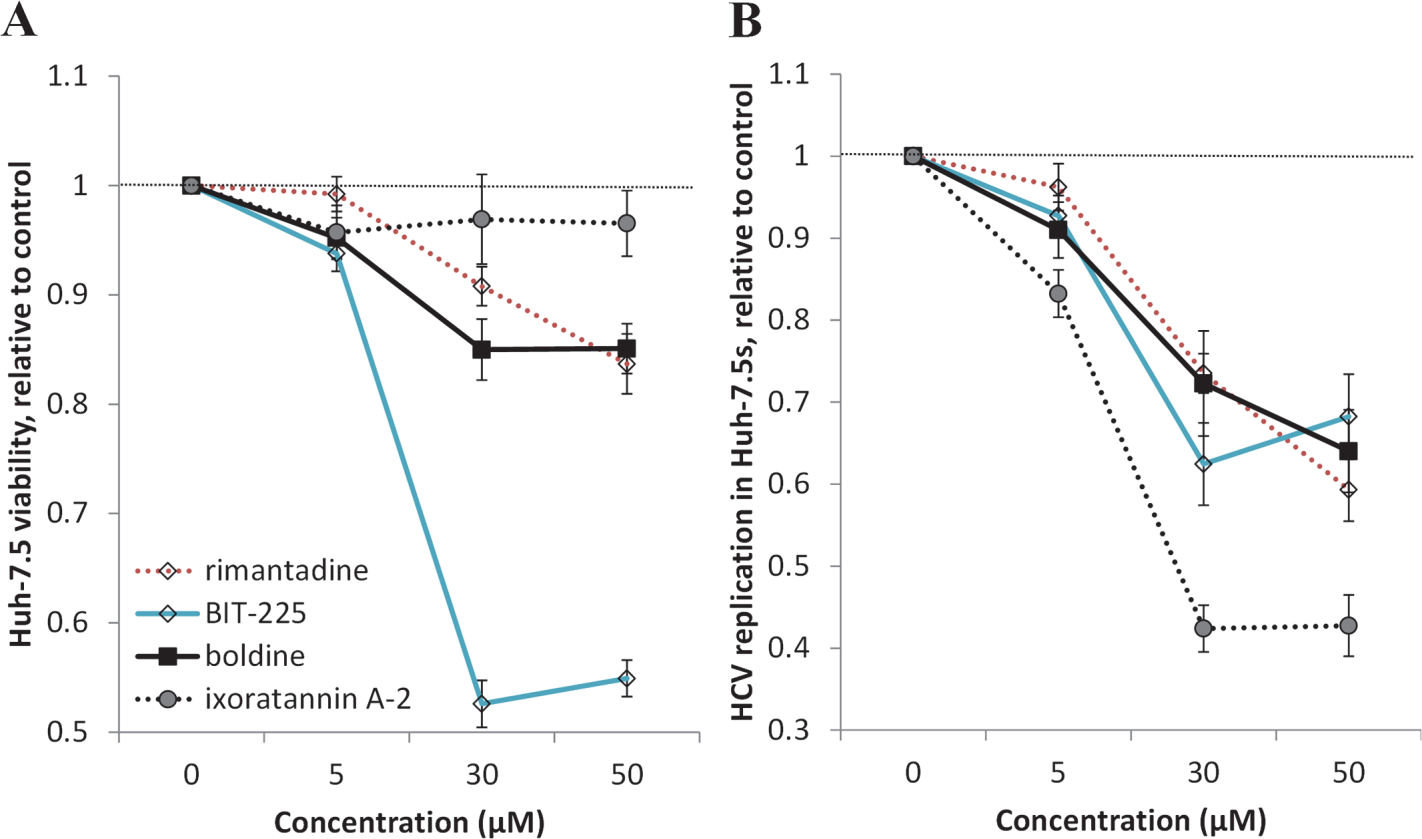
Effects of compounds on HCV replication. **A,** Cell viability at 72 h post-infection in the presence of compounds at defined concentrations. Data are normalized to the percent of viable cells in a HCV-infected culture plus 0.1% DMSO. **B,** HCV replication at 72 h post-infection in the presence of compounds. Data are normalized to percent HCV-infected cells plus 0.1% DMSO.

Interestingly, at 72 hours post-infection, no major cytotoxicity (defined here as at least 20% fewer gated cells compared to infected cells + 0.1% DMSO vehicle control) was observed at any concentration with the exception of BIT-225 (Fig. 6A) with a calculated CC_50_ of 52.9 μM. However, BIT-225 did inhibit HCV replication in surviving Huh-7.5 cells, with 37.5% inhibition at 30 μM (Fig. 6B). Boldine also inhibited HCV replication with efficacy similar to rimantadine [38], a previously-reported inhibitor of the HCV-encoded p7 ion channel and HCV replication (*e*.*g*., 36.0% and 40.7% inhibition at 50 μM for boldine and rimantadine, respectively). In contrast, ixoratannin A-2 was the most potent compound at all tested concentrations (Fig. 6B), with >50% inhibition of HCV at 30 μM and a calculated EC_50_ of 23.0 μM. The relatively low activity of control inhibitors like rimantadine and BIT-225 compared to previous reports [38, 39] are likely to reflect the stringent antiviral conditions of this assay, which features a 6 hour infection step before addition of drug in addition to a high multiplicity of infection (0.3).

## Discussion

The development of new therapeutics against HIV is necessary to counter the risks of resistance to established ARVs, particularly in countries with high disease burden and limited health care infrastructure. Here we used a common pharmacophore derived from a series of reported HIV-1 Vpu ion channel interactors [20] to perform a virtual screen of p-ANAPL molecules to identify compounds with potential new modes of action against HIV. Of eight molecules identified in this screen, two were observed to inhibit *in vitro* HIV-1 replication in a CD4+ T cell line as well as PBMCs. These compounds, ixoratannin A-2 and boldine, also had limited effects on cell viability when compared to their antiviral activities (*e*.*g*., selectivity indices (SIs) for ixoratannin A-2 and boldine in CEM-GXR cells were 1.7 and 4.1, respectively). Of the remaining six compounds, four did not inhibit HIV-1 replication in CEM-GXR cells at up to 100 μM, while two were highly cytotoxic (CC_50_s < 30 μM), which precluded detection of antiviral activity in this assay. We note that the limited anti-HIV activity of boldine and ixoratannin A-2, when compared to licensed ARVs, emphasizes that these compounds are at best starting points for new therapeutic development and are unlikely to exhibit any clinical benefit themselves. The limited anti-HIV activities of boldine and ixoratannin A-2 are also supported by experiments with HIV-1-infected PBMCs, where the antiviral activities of these compounds at 10 μM did not improve on the antiviral activity of 10 μM BIT-225, a molecule currently in phase I clinical trials [40].

Nevertheless, our results validate the strategy of using common pharmacophores from known and putative ion channel inhibitors to identify new antiviral compounds from natural products. Our results also highlight the p-ANAPL, a library of pure compounds derived from African plants with locally-reported medicinal properties [8, 9], as a viable source of therapeutically-relevant antiviral prototypes. As our initial screen here focused only on potential inhibitors of Vpu ion channel activity with structural similarity to known and putative Vpu inhibitors, additional compounds from p-ANAPL may also inhibit HIV-1 replication via similar or additional mechanisms. Assessment of these and other compounds for inhibitory activity against HIV and other pathogens is therefore underway.

Notably, we found that the Vpu inhibitor BIT-225, despite showing activity against HIV-1 and HCV replication in line with previous reports [19, 39], was highly toxic to CEM-GXR T cells (CC_50_ = 10.7 μM) and PBMCs (CC_50_ = 12.8 μM). The high cytotoxicity of BIT-225 in CEM-GXR cells precluded our ability to measure its antiviral activity in these cells, although antiviral activity of 10 μM BIT-225 was observed in PBMCs (31.8% inhibition). Our results contrast with previous studies that show minimal cytopathic effects of BIT-225 in monocyte derived macrophages (CC_50_s = 167–284 μM) and SupT1 and CEM-SS cells (CC_50_s = 32–67 μM) [36, 39]. However, bovine viral diarrhea virus-infected Madin-Darby bovine kidney cells were also reported to be sensitive to low levels of BIT-225 (CC_50_ = 11.6 μM) [41], and we also observed that Huh-7.5 cells (CC_50_ = 52.9 μM) are relatively tolerant to BIT-225 when compared to CEM-GXR cells and PBMCs. Thus, certain cell-types and cell lines, in addition to subsets of cells in PBMC cultures, likely differ in their sensitivity to BIT-225. Despite this, however, BIT-225 has advanced to early clinical trials to treat HCV and HIV-1 [41], where initial reports suggest that the disease-modifying activity of BIT-225 may be sufficient to overcome the potential adverse effects of cellular toxicity.

Of the two virtual screening hits found here, ixoratannin A-2 exhibited the lower selectivity index of blockade of HIV-1 replication over cytotoxicity in CEM-GXR cells (SI = 1.7). Ixoratannin A-2 is a doubly-linked, A-type proanthrocyanidin trimer isolated from the *Ixora coccinea* shrub of western Nigeria and traditionally used to treat infections, hypertension, menstrual irregularities, chronic ulcers, and skin diseases [21]. This compound contains three epicatechin units and has structural similarity to other flavanols and flavan-3-ols previously reported to have anti-HIV activity by targeting reverse transcriptase and integrase [2, 40, 42–43]. While we did not observe significant differences in the inhibition of EFV and RAL-resistant HIV-1 strains by ixoratannin A-2, except in some cases at 75 μM, these discrepancies could reflect differential modes of inhibition by ixoratannin A-2 compared to the mutant reverse transcriptase and integrase sequences examined here.

Evidence for Vpu as a possible target of ixoratannin A-2 comes from its modest (1.4-fold) inability to inhibit HIV-1 lacking a functional Vpu protein (HIV-1_Δvpu_). However, additional proof that ixoratannin A-2 directly blocks Vpu ion channel activity awaits detailed electrophysiology-based pharmacology studies in whole cells, which have been reported by some groups [14, 15, 44] but are disputed by others [16]. Moreover, as HIV-1_Δvpu_ resistance to ixoratannin A-2 was incomplete and occurred only at high concentrations (*i*.*e*., 75 μM), additional viral or host targets may exist. These possibilities require further study. Notably, ixoratannin A-2 inhibited multiple ARV-resistant HIV-1 strains with comparable EC_50_s to wild-type HIV-1_NL4-3_, indicating that it retains activity against HIV-1 harboring mutations that confer resistance to IND, EFV, or RAL, except at high concentrations. Thus ixoratannin A-2 and related compounds may indicate a potential therapeutic path toward overcoming resistance to EFV, IND, and RAL.

Boldine is an alkaloid aporphine that was originally isolated from the Chilean boldo tree (*Peumus boldus*) but is also found in many plants of African origin [45]. It is best characterized for its antioxidant, anti-inflammatory, and hepatoprotective properties and has been proposed as a potential therapy to treat cardiovascular, neurodegenerative, metabolic, and other inflammation-based diseases [46]. It is also reported to have antiplasmodial, anticancer, immunomodulatory and anticonvulsant properties [45]. Antiviral activity is also reported for other apomorphines, for example against HIV, poliovirus, rhinovirus, herpes simplex virus type 1, and influenza, although boldine itself was assessed in only a few of these studies, where no anti-polio virus or anti-herpes virus activity was observed [47–51].

While HIV-1_Δvpu_ was less responsive to treatment with boldine at multiple concentrations, these differences were not statistically significant and suggest that the discovery of boldine by virtual screening for similarity to other HIV-1 Vpu inhibitors may have been fortuitous. However, boldine has been reported to block hERG potassium channel currents, as measured by two electrode voltage clamp studies in *Xenopus* oocytes (IC_50_ = 19.3 μM) [52]. It is also an inhibitor of human 5-HT_3_ receptor ion channels, nicotinic acetylcholine receptors, and calcium channels at low micromolar concentrations [45, 53–55]. Thus, given its precedent as a promiscuous ion channel inhibitor, the anti-Vpu activity of boldine should also be confirmed in electrophysiological studies. We also found increased viral replication of all ARV-resistant viral strains, and HIV-1_PR+RT_ in particular, in the presence of boldine (*e*.*g*., 1.4-fold increased replication vs. HIV-1_NL4-3_). Thus, in addition to Vpu, HIV-1 protease, reverse transcriptase, and integrase are all potentially targets of boldine.

We also observed that ixoratannin A-2 and boldine inhibit HCV replication with similar or improved efficacy to rimantadine, an HCV ion channel inhibitor that is undergoing patient recruitment for a phase II clinical trial [56]. The activity of ixoratannin A-2 is also improved relative to BIT-225, which is also undergoing clinical trials for HCV [41]. Although recently-developed second-generation HCV protease inhibitors like Simeprevir display dramatically higher cure rates in HIV/HCV co-infected patients when compared to established pegylated interferon/ribavirin combination HCV therapies [57], morbidity in HIV/HCV co-infected patients—notably hepatocellular carcinoma and advanced liver damage—remains a concern [58]. Furthermore, it remains unclear whether these second-generation inhibitors can be effectively distributed to regions with limited healthcare infrastructure that has hampered access to ARVs. Therefore, ixoratannin A-2 and boldine could represent possible scaffolds for therapies to target HCV or HIV-1/HCV co-infection in the absence of established alternatives. These results also support the potential of ixoratannin A-2 and boldine for broad-spectrum antiviral activity, which should be assessed in additional viral systems.

No additional compounds were identified here to inhibit HIV-1. PA resembles stilbenoids isolated from the fruit of *Combretaceae hereroense* with reported antibacterial activity [22], and KA also has reported antibacterial, antifungal, antiprotozoal, antitumor, and antioxidant properties [23]. However, both of these compounds were cytotoxic in our viral assay (respective CC_50_s = 26.8 and 0.9 μM), making them poor candidates for further antiviral development at this stage. The potential antiviral activities of PA and KA will need to be assessed using alternative viral, enzymatic, or electrophysiological assays. We also did not observe antiviral activity for epicatechin gallate (ECG), which was previously reported to have anti-reverse transcriptase and integrase activities in enzymatic assays [2, 21, 40] but no antiviral activity in T cell lines [59]. Similarly, ellagic acid (EA) derivatives are reported to block HIV protease, integrase, and reverse transcriptase [60–62] but we did not observe these activities in our viral replication system. Finally, geshoidin, previously reported to inhibit glutathione transferases [24], and1-(3-acetyl-2,4-dihydroxy-6-((3,4,5,6-tetrahydroxytetrahydro-2H-pyran-2-yl)oxy)phenyl)-4,5-dihydroxy-2-methylanthracene-9,10-dione (2MA), which is structurally similar to compounds reported with tumor cytotoxic activity [25], also did not inhibit HIV-1 in our assay.

In conclusion, we report that pure compounds obtained from African medicinal plants, as part of the p-ANAPL, are a viable source of prototype antivirals. We identify new chemical scaffolds that may inform further development of natural product and/or synthetic HIV-1 inhibitors that act on ARV-resistant viral strains and alternative viral targets like Vpu, and we also provide new options for future anti-HIV and/or HCV therapies that might be suitable for local production.
